# TGF-β1 Facilitates TAp63α Protein Lysosomal Degradation to Promote Pancreatic Cancer Cell Migration

**DOI:** 10.3390/biology10070597

**Published:** 2021-06-28

**Authors:** Guohui Gao, Jie Chen, Dongbo Wang, Qiao Li, Xiaojiao Yang, Jindan Wang, Zhiyong Pan, Zhi-Xiong Jim Xiao, Yong Yi

**Affiliations:** 1 Center of Growth, Metabolism and Aging, Key Laboratory of Bio-Resource and Eco-Environment, Ministry of Education, College of Life Sciences, Sichuan University, Chengdu 610064, China; ggh1227@wmu.edu.cn (G.G.); 2018222040083@stu.scu.edu.cn (D.W.); jimzx@scu.edu.cn (Z.-X.J.X.); 2 Key Laboratory of Laboratory Medicine, School of Laboratory Medicine, Ministry of Education, Wenzhou Medical University, Wenzhou 325000, China; wjd@wmu.edu.cn (J.W.); pzy@wmu.edu.cn (Z.P.); 3 The First Clinical College, Wenzhou Medical University, Wenzhou 325000, China; chenjie_0810@wmu.edu.cn (J.C.); liqiao214@wmu.edu.cn (Q.L.); 4 School of Pharmacy, Wenzhou Medical University, Wenzhou 325000, China; yxj9597@wmu.edu.cn

**Keywords:** TGF-β1, TAp63α, p53, cell migration, pancreatic cancer

## Abstract

**Simple Summary:**

Numerous studies demonstrate that the activation of transforming growth factor-β (TGF-β) signaling is a critical driving force for promoting cancer cell migration and tumor metastasis. Our recent study indicates that TGF-β1 promotes FBXO3-mediated ΔNp63α protein degradation to facilitate cancer metastasis. In this study, we show that TGF-β1 can inhibit TAp63α protein stability in a lysosome-dependent, but canonical Smad pathway-independent manner, which leads to upregulation of p53-R248W expression, and consequently results in increased pancreatic cancer cell migration.

**Abstract:**

TGF-β signaling plays a pivotal role in promoting tumor cell migration and cancer metastasis. ΔNp63α and TAp63α are two major isoforms of p53-related p63 protein. Our recent study has shown that TGF-β1 promotes ΔNp63α protein degradation to facilitate cancer metastasis. However, whether TAp63α is involved in TGF-β1-induced cancer metastasis remains unclear. In this study, we show that, in human pancreatic cancer MIA PaCa-2 cells harboring p53-R248W allele, TGF-β1 can significantly inhibit TAp63α protein stability in a Smad pathway-independent manner. Lysosome inhibitor, chloroquine, but not proteasome inhibitor MG132, can rescue TGF-β1-induced downregulation of TAp63α protein. In addition, we show that either TGF-β1 treatment or silencing of TAp63α can dramatically increase migration of MIA PaCa-2 cells. Importantly, the restored expression of TAp63α can effectively block TGF-β1-induced migration of MIA PaCa-2 cells. Mechanistically, we show that TGF-β1 promotes TAp63α protein degradation, leading to upregulation of p53-R248W protein expression, and consequently resulting in elevated MIA PaCa-2 cell migration. Together, this study indicates that lysosomal degradation is an important way for regulating TAp63α protein fate and highlights that TGF-β1-TAp63α-mutant p53 axis is critically important in pancreatic cancer metastasis.

## 1. Introduction

TGF-β1 is a member of the TGF-β family which plays a pivotal role in a series of biological processes, including cell differentiation, proliferation, epithelial-mesenchymal transition (EMT), cell migration, tumor metastasis and immune escape [1,2,3]. As a ligand for TGF-β receptor, TGF-β1 binds to the TGF-β receptor and transduce signals through canonical Smad pathway or noncanonical Ras–Erk, PI3K/AKT and Rho-like GTPase pathways [4,5]. In the canonical Smad pathway, activated TGF-β receptor phosphorylates Smad2/Smad3, which then bind to Smad4 to form heteromeric Smad complexes translocated into the nucleus to transactivate downstream target genes [4,6,7]. Accumulating evidence indicates that TGF-β1 signaling can also execute its function through the regulation of protein stability. It is reported that TGF-β1 can promote iNOS protein degradation in a ubiquitin-proteasome-dependent manner during inflammatory process [8]. Furthermore, TGF-β1 can promote type I collagen protein lysosomal degradation [9]. Moreover, TGF-β1 can also facilitate MyD88 protein degradation by recruiting Smurf1 and Smurf2 [10].

p63,a p53 family member, which plays an important role in a variety of biological processes, including cell adhesion, proliferation, senescence, survival, tumor metastasis and embryonic development [11]. Derived from the alternative transcriptional start sites at the N termini and alternative splicing sites at the C termini, p63 gene encodes a series of protein isoforms [11]. ΔNp63α and TAp63α are two major isoforms of p63 family proteins. It has been well-documented that ΔNp63α is mainly expressed in epithelial cells and serves as an important tumor metastasis inhibitor [11]. The activation of PI3K/HER2/Ras can inhibit ΔNp63α gene transcription to promote cancer metastasis [12,13]. Oxidative stress suppresses cell motility and tumor metastasis via upregulation of ΔNp63α expression [14]. Our recent study demonstrates that TGF-β1 promotes FBXO3-mediated degradation of ΔNp63α to facilitate tumor metastasis [15]. On the other hand, TAp63α is mainly expressed in testicular tissues, ovarian, spermatocytes, and embryos [16,17,18,19,20,21]. It has been shown that TAp63α also plays a role in tumor metastasis. TAp63α can coordinately regulate Dicer and miR-130b to suppress tumor metastasis [22]. Furthermore, TAp63α can transcriptionally inhibit miR-19 to suppress tobacco smoke-induced lung cancer EMT [23]. Moreover, TAp63α can also transactivate miR-133b to inhibit colon cancer metastasis [24]. However, whether TAp63α is also involved in TGF-β1-induced cancer metastasis remains unclear.

In this paper, we show that TGF-β1 can significantly inhibit TAp63α protein stability in a lysosome-dependent manner, which leads to the upregulation of p53-R248W protein expression, and consequently results in increased MIA PaCa-2 cell migration.

## 2. Materials and Methods

### 2.1. Cell Culture and Drug Treatments

HEK293T (ATCC^®^ CRL-11268™) and MIA PaCa-2 (ATCC^®^ CRL-1420™, p53^R248W^) cells were purchased from American Type Culture Collection (ATCC, Manassas, VA, USA). MIA PaCa-2 and HEK293T cells were cultured in DMEM medium (GIBCO, Rockville, MD, USA) supplemented with 10% FBS (GIBCO), 100 μg/mL streptomycin (GIBCO) and 100 units/mL penicillin (GIBCO). Cells were grown in a humidified 37 °C incubator under a 5% CO_2_ atmosphere. Cells at 65–75% confluence were treated with TGF-β1 or an indicated chemical compound. MG132 (M8699) was purchased from MERCK (Darmstadt, Germany). Cycloheximide (CHX, ab120093) was purchased from Abcam (Cambridge, MA, USA). TGF-β1 (GF346) and chloroquine (CLQ, C6628) were purchased from Sigma (St Louis, MO, USA).

### 2.2. Plasmids and Lentiviral Infection

Expression plasmids include human TAp63α and TAp63γ. Lentiviruses were amplified by transfection of HEK293T cells with pMD2.G and psPAX2 packaging plasmids and Lentiviral-based expression plasmid using Lipofectamine 2000 (Invitrogen, Carlsbad, CA, USA). Viruses were collected at 60 h after transfection. Cells at 40–50% confluence were infected with recombinant Lentivirus encoding or an empty vector in the presence of 10 μg/mL polybrene, followed by 12 h. Lentiviral-based shRNAs specific for green fluorescent protein (GFP, GCATCAAGGTGAACTTCAA), p63 (1# CCGTTTCGTCAGAACACACAT; 2# GAGTGGAATGACTTCAACTTT) or p53 (GAGGGATGTTTGGGAGATGTA) were constructed as previous described [25].

### 2.3. Western Blot Analyses and Immunofluorescence Staining

Western blot analyses and immunofluorescence staining were performed as our previous described [13]. Antibodies for p63 (SC-8431), p53(SC-126) and Twist 1 (SC-15393) were purchased from Santa Cruz Biotech (Dallas, Texas, USA). Antibody for Snail1 (CST-3879), Smad3 (9523) and p-Smad3 (9520) were purchased from CST (Cambridge, MA, USA). Antibody for GAPDH (AF1186) was purchased from Beyotime (Shanghai, China).

### 2.4. Quantitative PCR (qPCR) Analyses

qPCR analyses were performed as our previous described [13]. qPCR primer specifics for TAp63 (F: CCCAGAGTCTTCCAGCATA; R: TTTTCGGAAGGTTCATCCAC), p53 (F: CCAACAACACCAGCTCCTCT; R: CCTCATTCAGCTCTCGGAAC) and GAPDH (F: GAGTCAACGGATTTGGTCGT; R: TTGATTTTGGAGGGATCTCG) were used.

### 2.5. Transwell Assay for Cell Migration

Cell migration was measured as our previous described [26]. In briefly, MIA PaCa-2 cells (3 × 10^5^) grown in serum-free DMEM medium were seeded into transwell inner chamber. The outer chamber was filled with regular DMEM medium. Cells were incubated for 24 h. Non-migrating cells were carefully removed and migrating cells were stained with 0.1% crystal violet for 10–15 min at room temperature. Cells were photographed using a Nikon light microscope. At least 100 cells from five random fields were counted.

### 2.6. Statistical Analysis

GraphPad Prism 6.0 (GraphPad Software Inc., San Diego, CA, USA) was used to statistical analysis. All experiments were performed at least three times in triplicate. Student’s *t*-test was used to assess significance.

## 3. Results

### 3.1. TGF-β1 Promotes TAp63α Protein Degradation in A Lysosome-Dependent Manner

**Figure 1:**
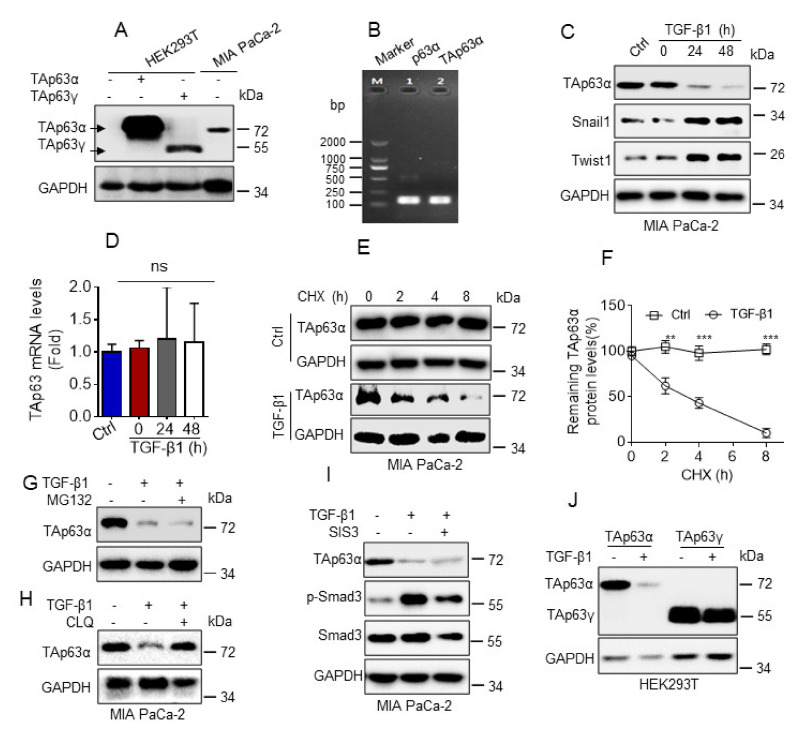
TGF-β1 promotes TAp63α protein degradation in a lysosome dependent manner. (**A**) HEK293Tcells were transiently transfected with a vector control, TAp63α orTAp63γ. Cells were subjected to western blot analyses. (**B**) Reverse transcription PCR was performed to examine the mRNA transcripts of p63 in MIA PaCa-2 cells. (**C**,**D**) MIA PaCa-2 cells were treated or untreated with TGF-β1 (5 ng/mL) for an indicated time point under serum-free condition. Cells were subjected to western blot (**C**) or qPCR (**D**) analyses. (**E**,**F**) MIA PaCa-2 cells were treated with cycloheximide (CHX, 50 μg/mL) in the presence or absence of TGF-β1 (5 ng/mL) for the indicated time intervals under serum-free condition. Cells were subjected to western blot analyses (**E**). Western blot images were analyzed using Image Lab software, and TAp63α protein half-life was plotted as shown (**F**). (**G**,**H**) MIA PaCa-2 cells were treated or untreated with TGF-β1 (5 ng/mL) for 12 h prior to treatment with MG132 (10 μM) (**G**) or chloroquine (CLQ, 50 μM) (**H**) for 12 h under serum-free condition. Cells were subjected to western blot analyses. (**I**) MIA PaCa-2 cells were treated or untreated with 5 ng/mL TGF-β1 in the presence or absence of 3 μM SIS3 for 24 h under serum-free condition. Cells were subjected to western blot analyses. (**J**) HEK293T cells were transient transfected with TAp63α or TAp63γ. Cells were treated with or without 5 ng/mL TGF-β1 for 24 h under serum-free condition. Cells were subjected to western blot analyses. Data are presented as means ± s.d. **, *p* < 0.01; ***, *p* < 0.001; ns, no significance. Original images supporting all western blot results reported in Figures S1–S16.

TAp63α and ΔNp63α are two major isoforms of p63 protein. Our recent study demonstrates that TGF-β1 promotes FBXO3-mediated ΔNp63α protein proteasomal degradation to facilitate cancer metastasis [15]. However, whether TAp63α also plays a role in TGF-β1-induced tumor metastasis remains unclear. To explore this issue, we explored the effects of TGF-β1 on TAp63α expression. Human pancreatic cancer MIA PaCa-2 cells primarily expressed TAp63α isoform, as evidenced by western blot and RT-PCR (reverse transcriptional PCR) analyses (Figure 1A,B). As shown in Figure 1C, TGF-β1 treatment significantly upregulated epithelial-mesenchymal transition (EMT) markers Twist1 and Snail1 expression, as expected. Notably, TGF-β1 markedly inhibited TAp63α protein expression. Next, we explored the molecular basis by which TGF-β1 inhibits TAp63α expression. As shown in Figure 1D, TGF-β1 treatment had no effects on TAp63α mRNA levels in MIA PaCa-2 cells, suggesting TGF-β1 can’t affect TAp63α transcription. By contrast, TGF-β1 treatment significantly shortened TAp63α protein half-life in MIA PaCa-2 cells (Figure 1E,F). It is well-known that ubiquitin-proteasome and autophagy/lysosome are two major systems for protein degradation. To explore the role of proteasome and lysosome in TGF-β1-induced TAp63α protein degradation, we used proteasome inhibitor MG132 or lysosome inhibitor chloroquine (CLQ) to treat MIA PaCa-2 cells. As shown in Figure 1G, proteasome inhibitor MG132 treatment had no effect on TGF-β1-induced downregulation of TAp63α protein expression. However, TGF-β1-induced downregulation of TAp63α protein level was completely blocked by lysosome inhibitor chloroquine (CLQ) (Figure 1H). Next, we investigated whether TGF-β1 promotes TAp63α lysosomal degradation is dependent of canonical Smad pathway. As shown in Figure 1I, treatment with Smad3 inhibitor SIS3 had no effects on TGF-β1-induced downregulation of TAp63α. Moreover, we found that TGF-β1 had little effects on TAp63γ protein expression (Figure 1J). Together, these results demonstrate that TGF-β1 specifically promotes TAp63α protein lysosomal degradation in a canonical Smad-independent manner in MIA PaCa-2 cells.

### 3.2. TGF-β1 Inhibits TAp63α to Promote Pancreatic Cancer Cell Migration

**Figure 2:**
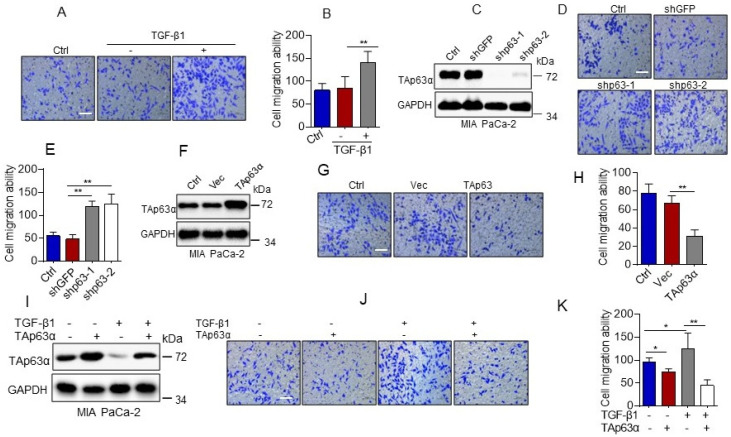
TGF-β1 promotes pancreatic cancer cell migration by suppressing TAp63α. (**A**,**B**) MIA PaCa-2 cells were treated or untreated with TGF-β1 (5 ng/mL) for 36 h. Cell motility was examined by transwell assays. (**C**–**E**) MIA PaCa-2 cells stably expressing shGFP, shp63-1 or shp63-2 were subjected to Western blot analyses (**C**) or transwell assay for cell motility (**D**,**E**). (**F**–**H**) MIA PaCa-2 cells stably expressing a vector control (Vec) or TAp63α were subjected to Western blot analyses (**F**) or transwell assay for cell motility (**G**,**H**). (**I**–**K**) MIA PaCa-2 cells stably expressing TAp63α or Vec were treated or untreated with 5 ng/mL TGF-β1 for 36 h. Cells were subjected to Western blot analyses (**I**) or transwell assay for cell motility (**J**,**K**). Data are presented as means ± s.d. **, *p* < 0.01; *, *p* < 0.05. Original images supporting all western blot results reported in Figures S1–S16.

Our abovementioned data show that TGF-β1 promotes TAp63α protein lysosomal degradation. Since TAp63α also serves as a key tumor metastasis suppressor, we thus, asked whether TGF-β1-induced downregulation of TAp63α contributes to pancreatic cancer cell migration. As shown in Figure 2A,B, TGF-β1 significantly increased MIA PaCa-2 cell migration, as evidenced by transwell assays. In addition, silencing of p63 by short hairpin RNA (shRNA) led to significantly increased MIA PaCa-2 cell migration (Figure 2C,E). Conversely, ectopic expression of TAp63α suppressed MIA PaCa-2 cell migration (Figure 2F–H). Importantly, TGF-β1-induced upregulation of MIA PaCa-2 cell migration was totally blocked by ectopic expression of TAp63α (Figure 2I–K). Together, these results indicate that downregulation of TAp63α plays a causative role in TGF-β1-induced MIA PaCa-2 cell migration.

### 3.3. TGF-β1-Induced Downregulation of TAp63α Upregulates Mutant p53 Expression to Promote Pancreatic Cancer Cell Migration

**Figure 3:**
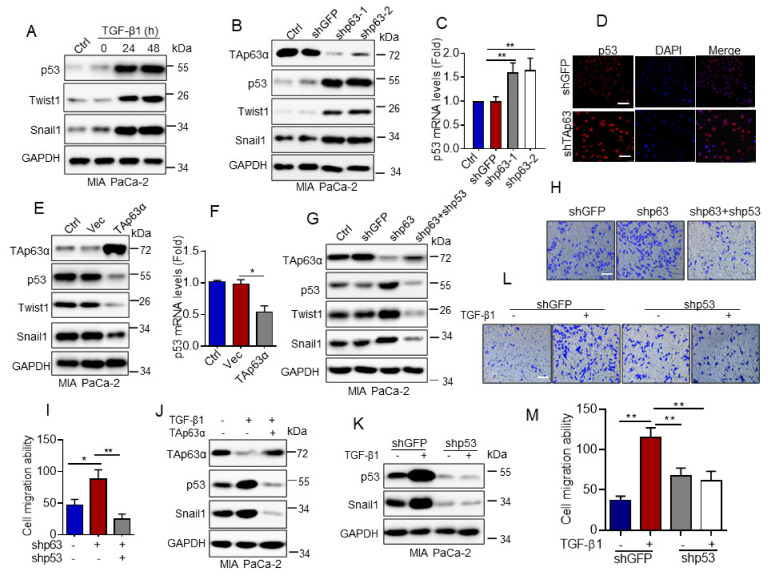
TGF-β1-induced downregulation of TAp63α promotes MIA PaCa-2 cell motility via upregulation of mutant p53. (**A**) MIA PaCa-2 cells were treated or untreated with TGF-β1 (5 ng/mL) for an indicated time point under serum-free condition. Cells were subjected to western blot analyses. (**B**–**D**) MIA PaCa-2 cells stably expressing shGFP, shp63-1 or shp63-2 were subjected to western blot analyses (**B**), qPCR analyses (**C**) or to immuno-fluorescent staining for p53 (**D**). (**E**,**F**) MIA PaCa-2 cells stably expressing TAp63α or Vec were subjected to western blot (**E**) or qPCR analyses (**F**). (**G**–**I**) MIA PaCa-2-shp63 cells stably expressing shp53 were subjected to western blot analyses (**G**) or transwell assay for cell motility (**H**,**I**). (**J**) MIA PaCa-2 cells stably expressing TAp63α or Vec were treated or untreated with 5 ng/mL TGF-β1 for 36 h. Cells were subjected to western blot analyses. (**K**–**M**) MIA PaCa-2 cells stably expressing shp53 or shGFP were treated or untreated with TGF-β1 (5 ng/mL) for 36 h. Cells were subjected to western blot analyses (**K**) or transwell assay for cell motility (**L**,**M**). Data are presented as means ± s.d. **, *p* < 0.01; *, *p* < 0.05. Original images supporting all western blot results reported in Figures S1–S16.

Next, we examined the molecular mechanism by which TAp63α regulates MIA PaCa-2 cell migration. It has been documented that cancer-associated p53 hot-spot mutants, including R175H, R273H and R248W, possesses gain of function in promoting cancer metastasis [26]. Since MIA PaCa-2 cells carry a p53-R248W mutation [27]. We asked whether TGF-β1 affects p53-R248W protein expression in MIA PaCa-2 cells. As shown in Figure 3A, TGF-β1 significantly upregulated expression of EMT markers Twist1 and Snail1, as expected. It markedly elevated p53-R248W protein expression. Furthermore, knockdown of TAp63α by shRNAs dramatically increased p53-R248W protein expression, concomitant with increased expression of Twist1 and Snail1 proteins in MIA PaCa-2 cells (Figure 3B). qPCR analyses showed that silencing of TAp63α significantly upregulated expression of p53-R248W mRNA (Figure 3C). Immunofluorescence staining assay showed that knockdown of TAp63α dramatically led to accumulation of p53-R248W in the nucleus (Figure 3D). On the other hand, ectopic expression of TAp63α significantly inhibited expression of both p53-R248W mRNA and protein, concomitant with reduced expression of Twist1 and Snail1 proteins in MIA PaCa-2 cells (Figure 3E,F). Importantly, knockdown of TAp63α-induced upregulation of Snail1 and twist1 protein expression and increased MIA PaCa-2 cell migration were totally reversed by simultaneous knockdown of p53-R248W (Figure 3G,I). Next, we investigated whether TGF-β1 promotes p53-R248W expression via inhibiting TAp63α. As shown in Figure 3J, ectopic expression of TAp63α can totally inhibit TGF-β1-induced upregulation of p53-R248W and Snail1 protein expression. In addition, TGF-β1-induced MIA PaCa-2 cell migration can be completely suppressed by silencing of p53-R248W expression, consistent with ectopic expression of TAp63α (Figure 2I,K and Figure 3K,M). Together, these results indicate that TGF-β1 up-regulates p53-R248W expression via suppression of TAp63α, thereby facilitating MIA PaCa-2 cell migration.

## 4. Discussion

**Figure 4:**
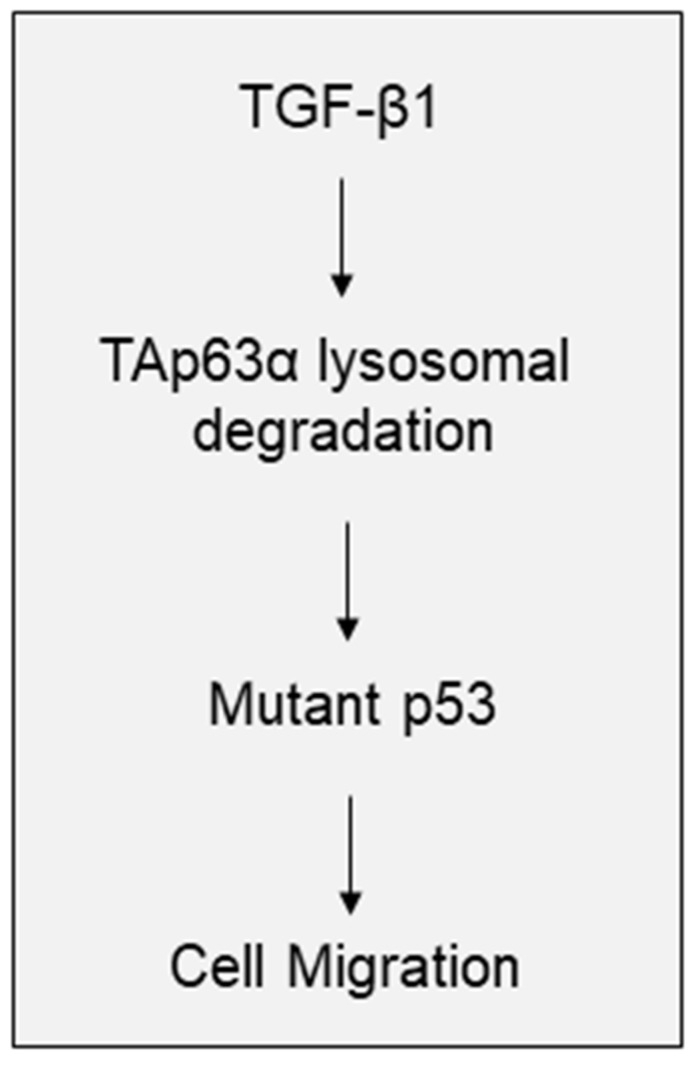
A model depicts the role of TAp63α in TGF-β1-induced pancreatic cancer cell motility. TGF-β1 promotes TAp63α lysosomal degradation, which leads to the upregulation of mutant p53 expression, consequently results in pancreatic cell migration.

TGF-β signaling plays a pivotal in the regulation of EMT, cell migration and tumor metastasis [1,2,3]. It has been documented that the activation of TGF-β signaling can promote expression of Snail, Twist1, Slug, and ZEB1, which in turn, regulates the expression of N-cadherin, Vimentin and, E-cadherin, proteins critically involved in EMT [28]. Moreover, TGF-β1 can activate PI3K/AKT/mTOR signaling or promote expression of EGFR and FosB to facilitate cancer cell motility, invasion and tumor metastasis [29,30]. Furthermore, TGF-β1 has been shown to promote mutant-p53/Smad complex formation, which antagonizes TAp63 transcriptional activity to facilitate cancer metastasis [31]. Our recent study demonstrates that TGF-β1 promotes FBXO3-mediated ΔNp63α protein degradation to enhance tumor metastasis [15]. In here, we show that TGF-β1 can inhibit TAp63α protein stability in a lysosome-dependent, but canonical Smad pathway-independent manner, leading to upregulation of p53-R248W expression, and consequently resulting in increased MIA PaCa-2 cell migration (Figure 4).

Accumulating evidence indicate that TGF-β signaling regulates a series of genes transcription through canonical Smad pathway or noncanonical Ras–Erk, PI3K/AKT, and Rho-like GTPase pathways [4,5]. Furthermore, it is reported that physiologic levels of TGF-β1 can stimulate paxillin mRNA translation [32]. In recent years, lots of researches demonstrated that TGF-β signaling also can regulate protein degradation. TGF-β1 can promote iNOS and MyD88 protein degradation in a proteasome-dependent manner [8,10]. Furthermore, TGF-β1 can promote type I collagen protein lysosomal degradation [9]. Our recent study demonstrates that TGF-β1 promotes ΔNp63α protein proteasomal degradation [15]. In this study, we show that TGF-β1 can facilitate TAp63α protein degradation in lysosome-dependent manner. Therefore, TGF-β signaling regulates protein expression in multiple levels, including transcription, translation and protein degradation.

Understanding how TGF-β1 facilitates TAp63α protein lysosomal degradation is not yet clear. It has been reported that the chaperone-mediated autophagy (CMA) is one of the intracellular proteolytic machineries for selective protein degradation within lysosome [33]. In the CMA process, CMA substate interacts with heat shock cognate protein 70 (Hsc70), which carries CMA substate to lysosome to degradation via lysosomal membrane protein type 2a (LAMP2A) [33]. Since TGF-β can facilitate Hsc70 and Smad2/3 interaction [34], it is plausible that TGF-β1 may promote TAp63α and Hsc70 interaction, resulting in TAp63α protein degradation in a CMA-dependent manner, a possibility needs to be further investigated.

While ΔNp63α is mainly expressed in epidermis [11], the expression of TAp63α in epidermis is extremely low [35]. Interestingly, it has been shown that the expression of TGF-β1 is high in epidermis [36]. In this study, we show that TGF-β1 inhibit TAp63α expression. Whether high levels of TGF-β1 contribute to low levels of TAp63α expression in epidermis remains to be seen.

## 5. Conclusions

Numerous studies demonstrate that TGF-β signaling plays an important role in promoting cancer cell migration and tumor metastasis. In this study, we show that TGF-β1 can specifically inhibit TAp63α, a p53 family member, protein stability in a lysosome-dependent, but canonical Smad pathway-independent, manner, which leads to upregulation of p53-R248W protein expression, and consequently results in pancreatic cancer cell migration. Our study reveals a new molecular mechanism by which TGF-β1 promotes cancer cell migration and demonstrates that lysosomal degradation is a novel way to regulate TAp63α protein fate.

## Supplementary Materials

The following are available online at https://www.mdpi.com/2079-7737/10/7/597/s1, Figures S1–S16. The experimental samples, loading order, molecular weight markers, and protein quantitation are indicated. Original images supporting all western blot results reported in Figures S1–S16.

## Abbreviations

CLQ	chloroquine
EMT	epithelial-mesenchymal transition
CMA	chaperone-mediated autophagy
