# Correction: Gao et al. TGF-β1 Facilitates TAp63α Protein Lysosomal Degradation to Promote Pancreatic Cancer Cell Migration. *Biology* 2021, *10*, 597

**DOI:** 10.3390/biology11111558

**Published:** 2022-10-24

**Authors:** Guohui Gao, Jie Chen, Dongbo Wang, Qiao Li, Xiaojiao Yang, Jindan Wang, Zhiyong Pan, Zhi-Xiong Jim Xiao, Yong Yi

**Affiliations:** 1 Center of Growth, Metabolism and Aging, Key Laboratory of Bio-Resource and Eco-Environment, Ministry of Education, College of Life Sciences, Sichuan University, Chengdu 610064, China; 2 Key Laboratory of Laboratory Medicine, School of Laboratory Medicine, Ministry of Education, Wenzhou Medical University, Wenzhou 325000, China; 3 The First Clinical College, Wenzhou Medical University, Wenzhou 325000, China; 4 School of Pharmacy, Wenzhou Medical University, Wenzhou 325000, China

## Error in Figure/Table

In the original publication [1], there was a mistake in **Figure 2** as published. **In Figure 2D, the transwell image shown in panel shp63-2 is incorrect.** The corrected Figure 2 appears below. The authors state that the scientific conclusions are unaffected. This correction was approved by the Academic Editor. The original publication has also been updated.

## Figures and Tables

**Figure 2:**
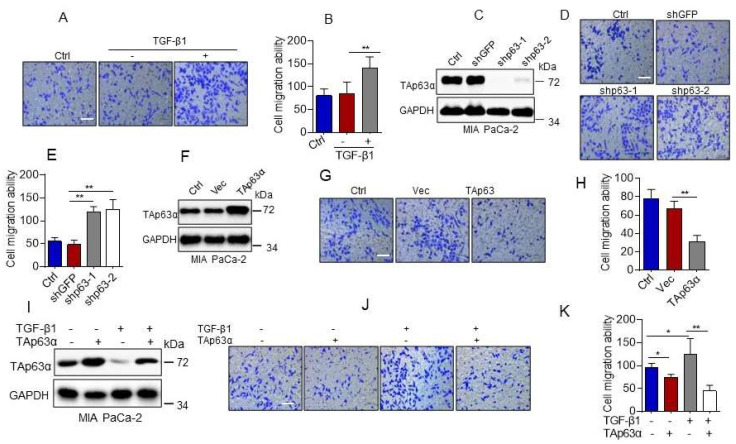
TGF-β1 promotes pancreatic cancer cell migration by suppressing TAp63α. (**A**,**B**) MIA PaCa-2 cells were treated or untreated with TGF-β1 (5 ng/mL) for 36 h. Cell motility was examined by transwell assays. (**C**–**E**) MIA PaCa-2 cells stably expressing shGFP, shp63-1 or shp63-2 were subjected to Western blot analyses (**C**) or transwell assay for cell motility (**D**,**E**). (**F**–**H**) MIA PaCa-2 cells stably expressing a vector control (Vec) or TAp63α were subjected to Western blot analyses (**F**) or transwell assay for cell motility (**G**,**H**). (**I**–**K**) MIA PaCa-2 cells stably expressing TAp63α or Vec were treated or untreated with 5 ng/mL TGF-β1 for 36 h. Cells were subjected to Western blot analyses (**I**) or transwell assay for cell motility (**J**,**K**). Data are presented as means ± s.d. **, *p* < 0.01; *, *p* < 0.05. Original images supporting all western blot results reported in Figures S1–S16.
