# Supplementary material for: TGF-β1 Facilitates TAp63α Protein Lysosomal Degradation to Promote Pancreatic Cancer Cell Migration
Source: Biology (Basel). 2021 Jun 28;10(7):597. doi: 10.3390/biology10070597 (PMC8301043; doi:10.3390/biology10070597)
Supplement: Supplementary file 1 [file biology-10-00597-s001.zip › biology-1202996-supplementary.pdf]

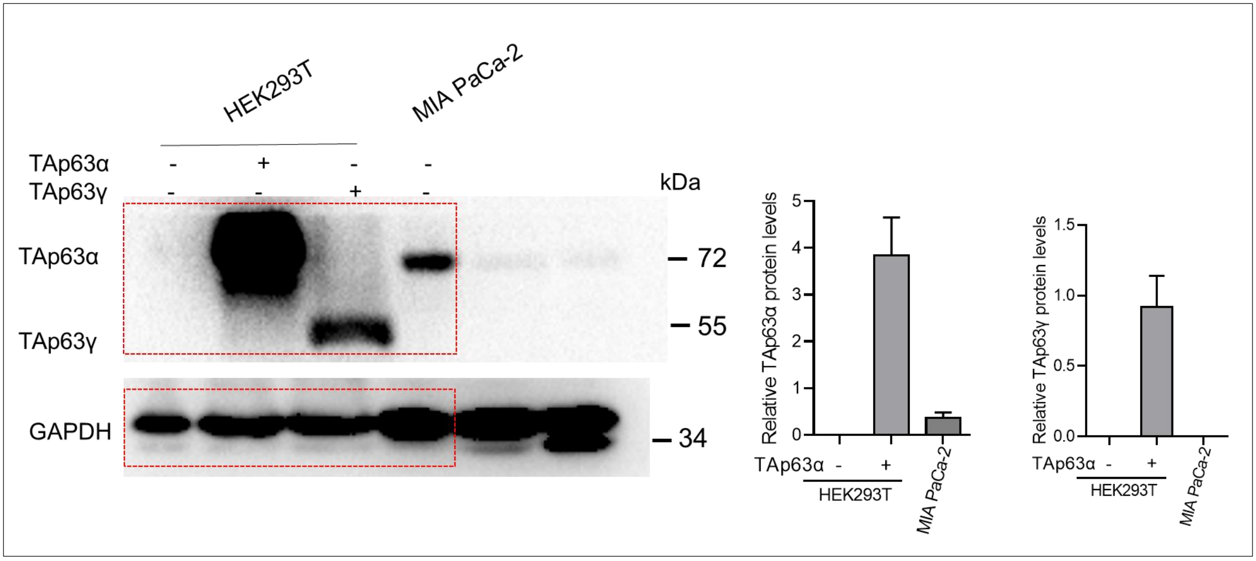


**Figure S1.** Human pancreatic cancer MIA PaCa-2 cells primarily expressed TAp63*α* isoform.

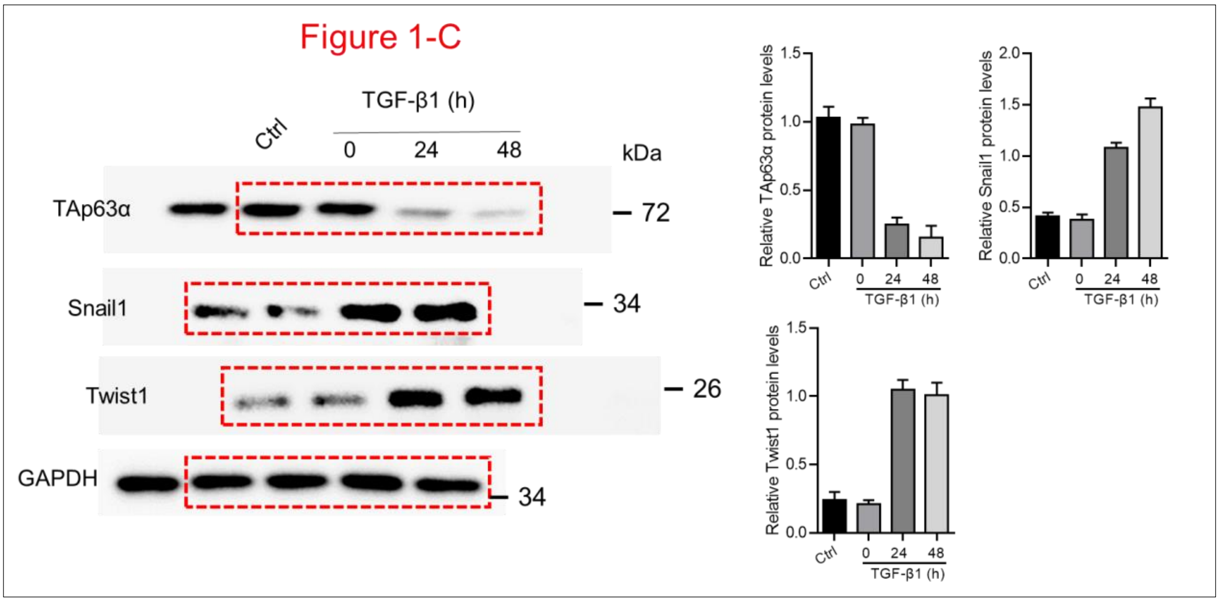


**Figure S2.** TGF-β1 treatment significantly upregulated epithelial-mesenchymal transition (EMT) markers Twist1 and Snail1 expression and markedly inhibited TAp63*α* protein expression.

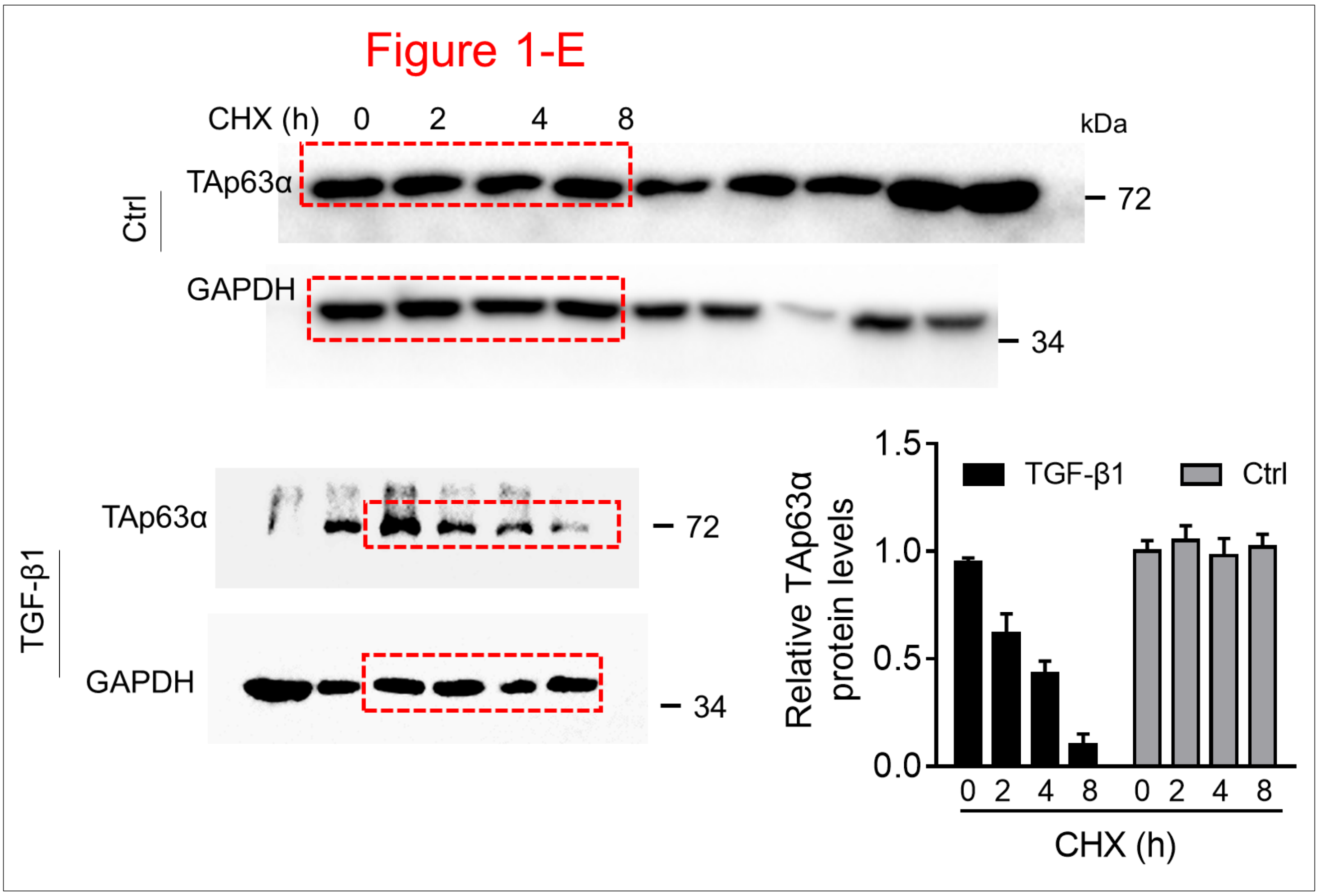


**Figure S3.** TGF-β1 treatment significantly shortened TAp63α protein half-life.

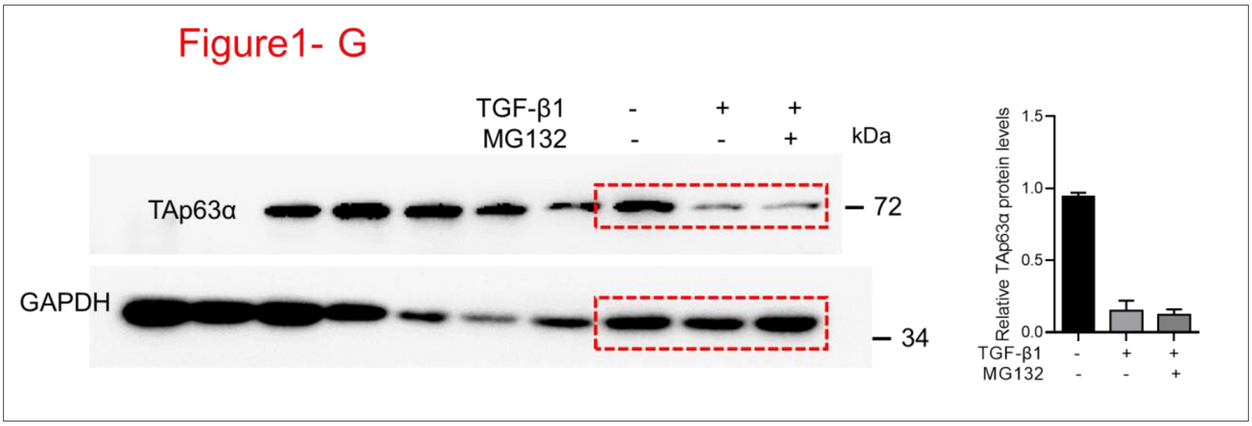


**Figure S4.** Proteasome inhibitor MG132 treatment had no effect on TGF-β1-induced downregulation of TAp63α protein expression.

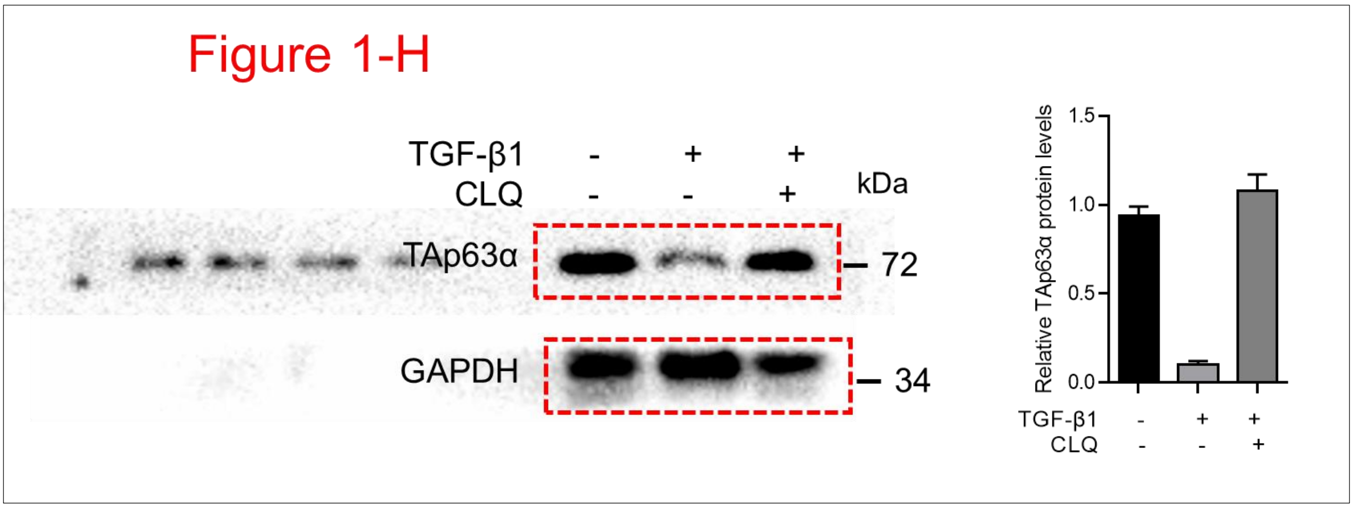


**Figure S5.** TGF-β1-induced downregulation of TAp63α protein level was completely blocked by lysosome inhibitor chloroquine (CLQ).

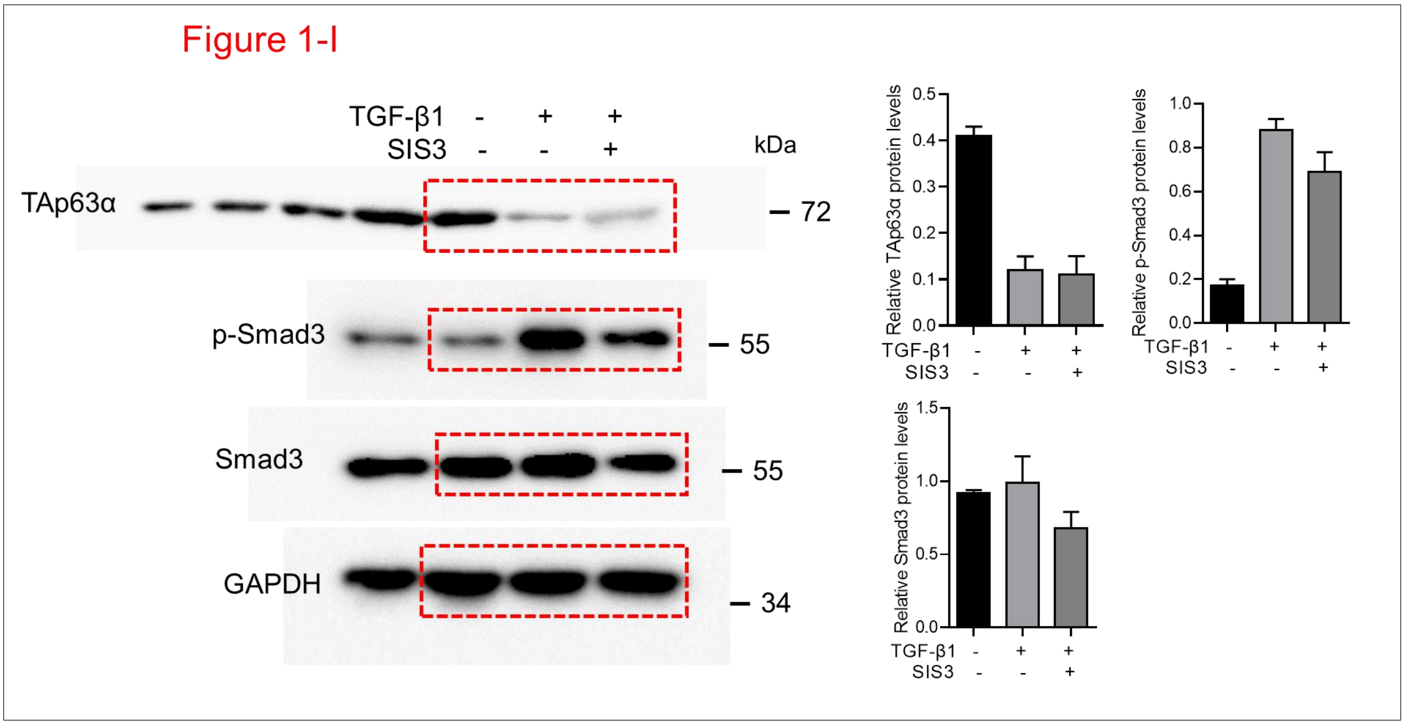


**Figure S6.** Treatment with Smad3 inhibitor SIS3 had no effects on TGF-β1-induced downregulation of TAp63α.

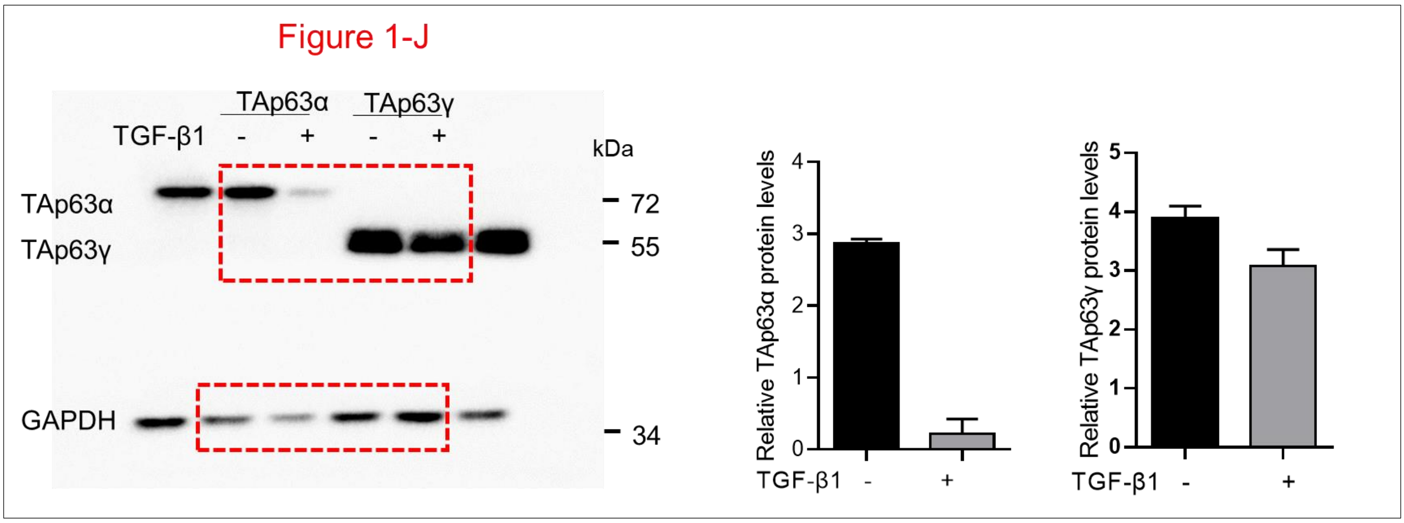


**Figure S7.** TGF-β1 treatment markedly inhibited TAp63α protein expression, but it had little effects on TAp63γ protein expression.

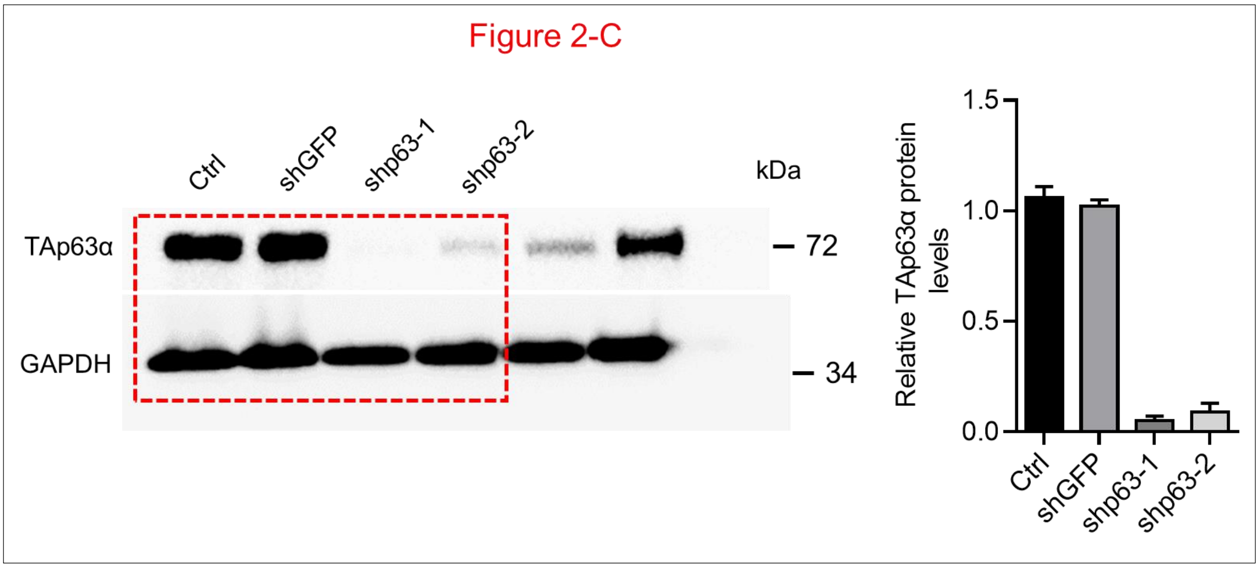


**Figure S8.** Silencing of TAp63α by short hairpin RNA in MIA PaCa-2 cells.

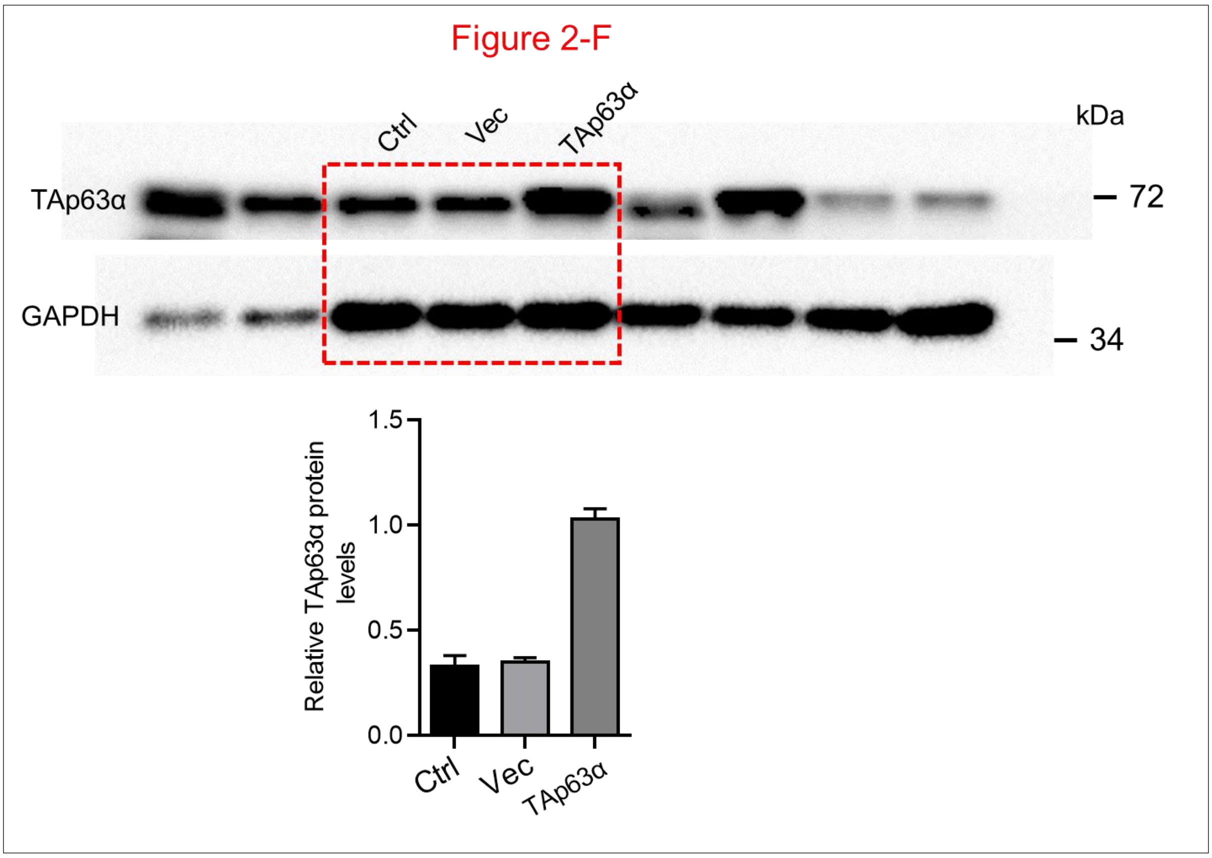


**Figure S9.** Ectopic expression of TAp63*α* in MIA PaCa-2 cells.

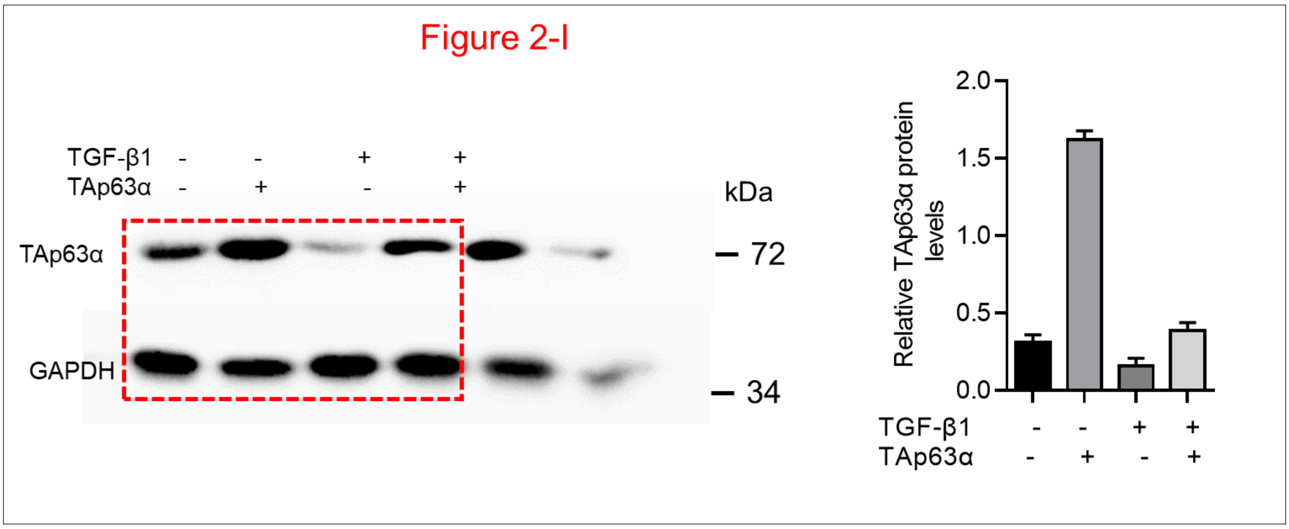


**Figure S10.** MIA PaCa-2 cells stably expressing TAp63*α* were treated or untreated with TGF-β1.

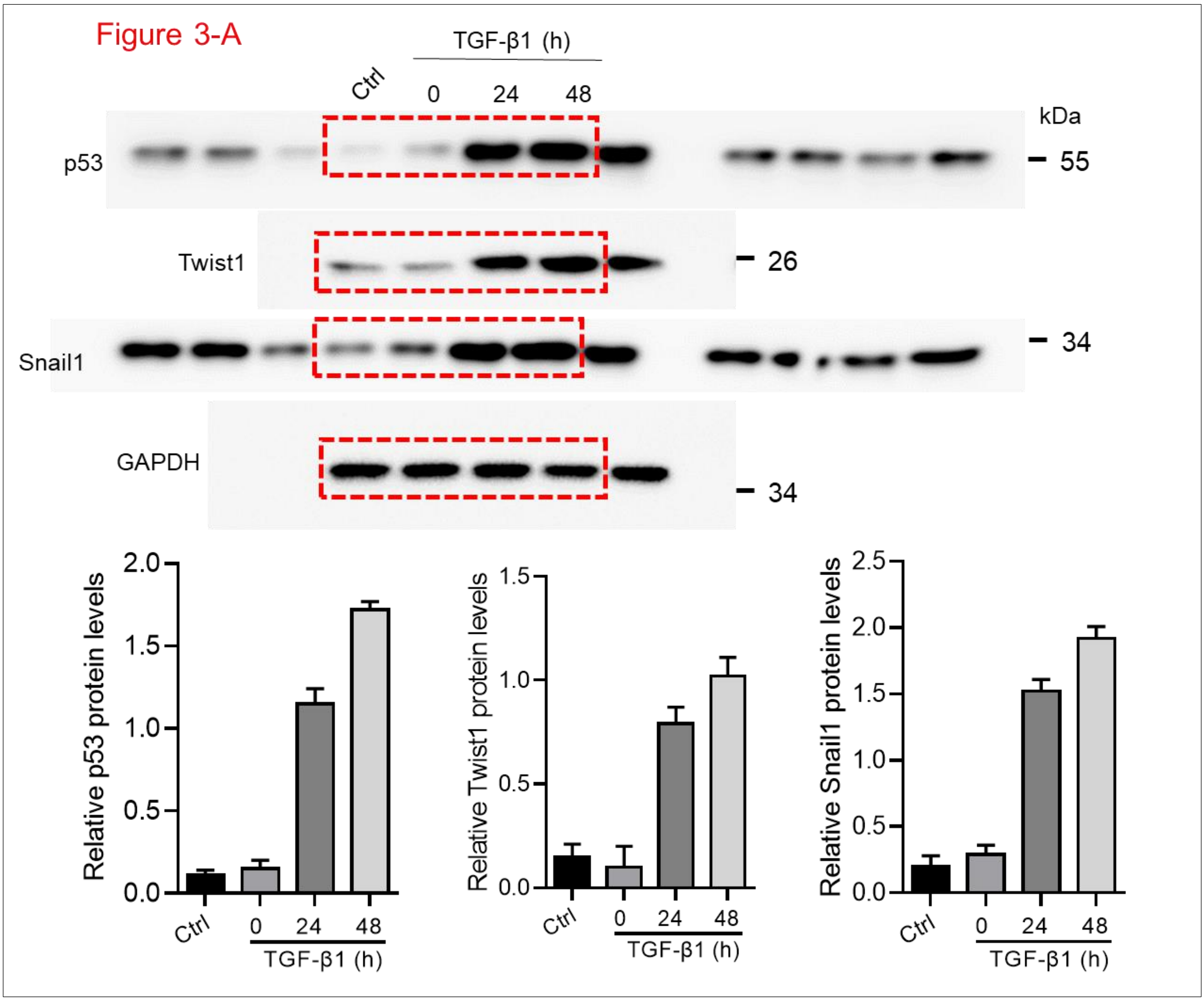


**Figure S11.** TGF-β1 significantly upregulated expression of EMT markers Twist1 and Snail1 and markedly elevated p53-R248W protein expression.

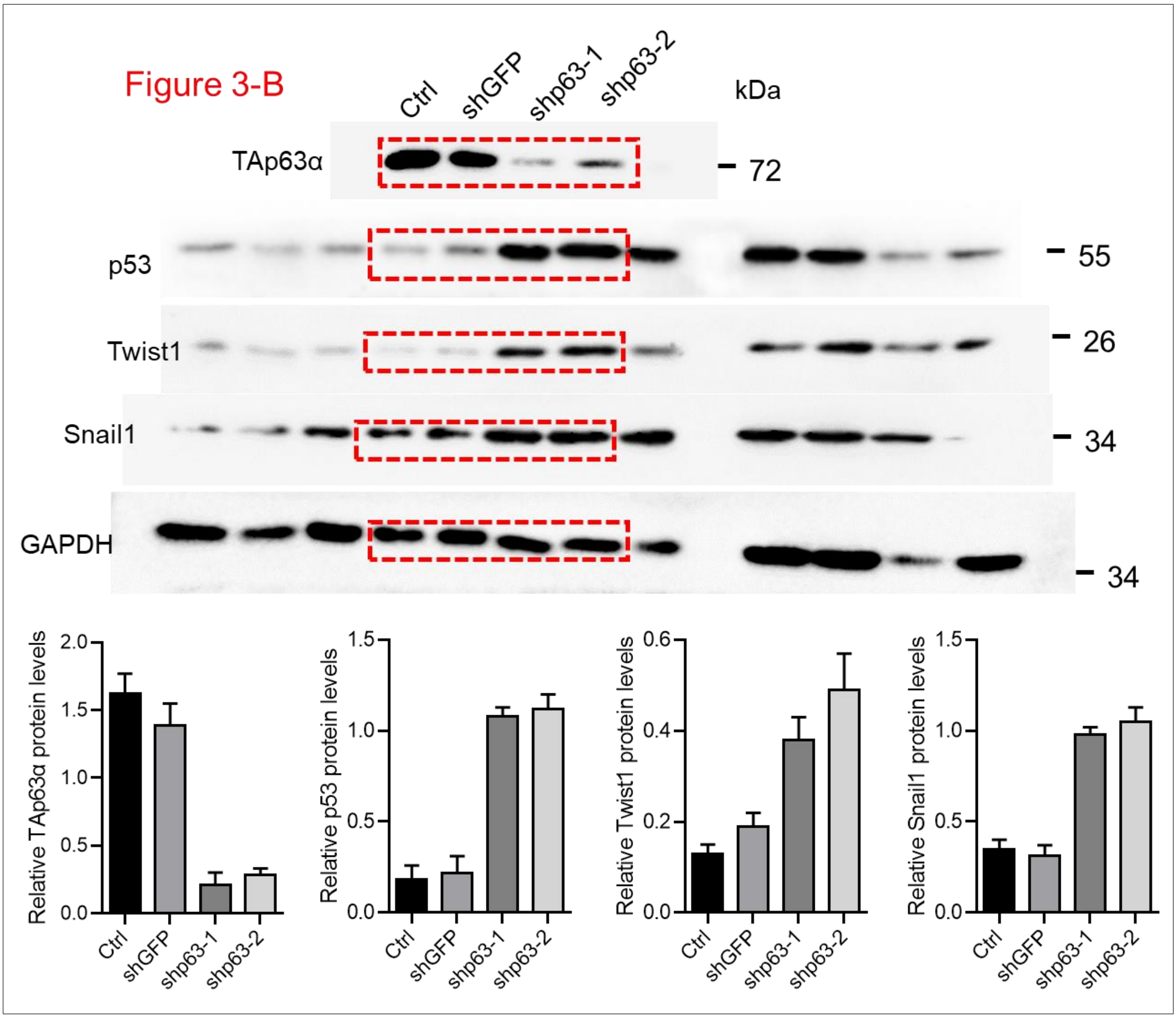


**Figure S12.** knockdown of TAp63*α* dramatically increased p53-R248W protein expression, concomitant with increased expression of Twist1 and Snail1 proteins.

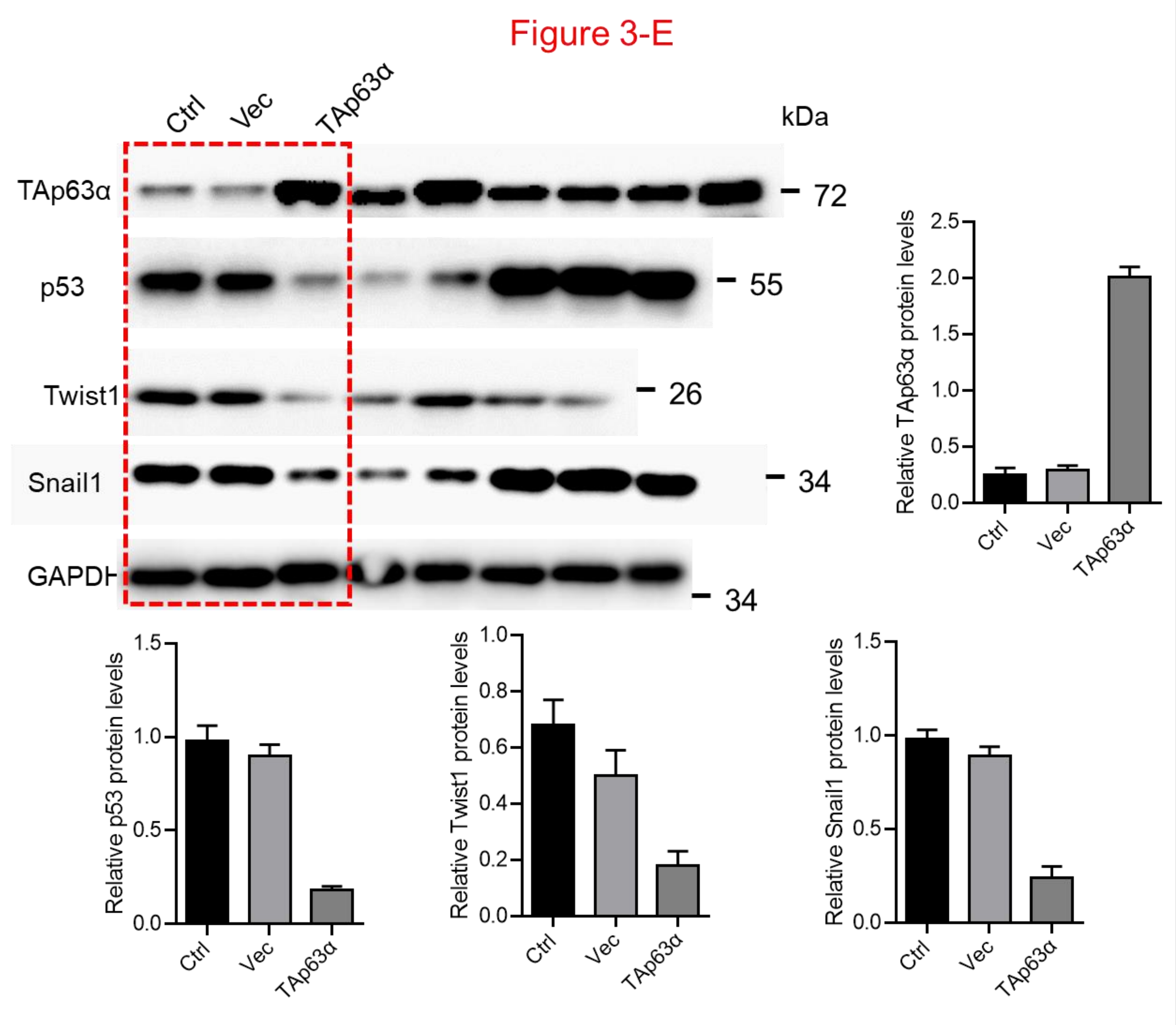


**Figure S13.** Ectopic expression of TAp63α significantly inhibited expression of p53-R248W protein, concomitant with reduced expression of Twist1 and Snail1 proteins.

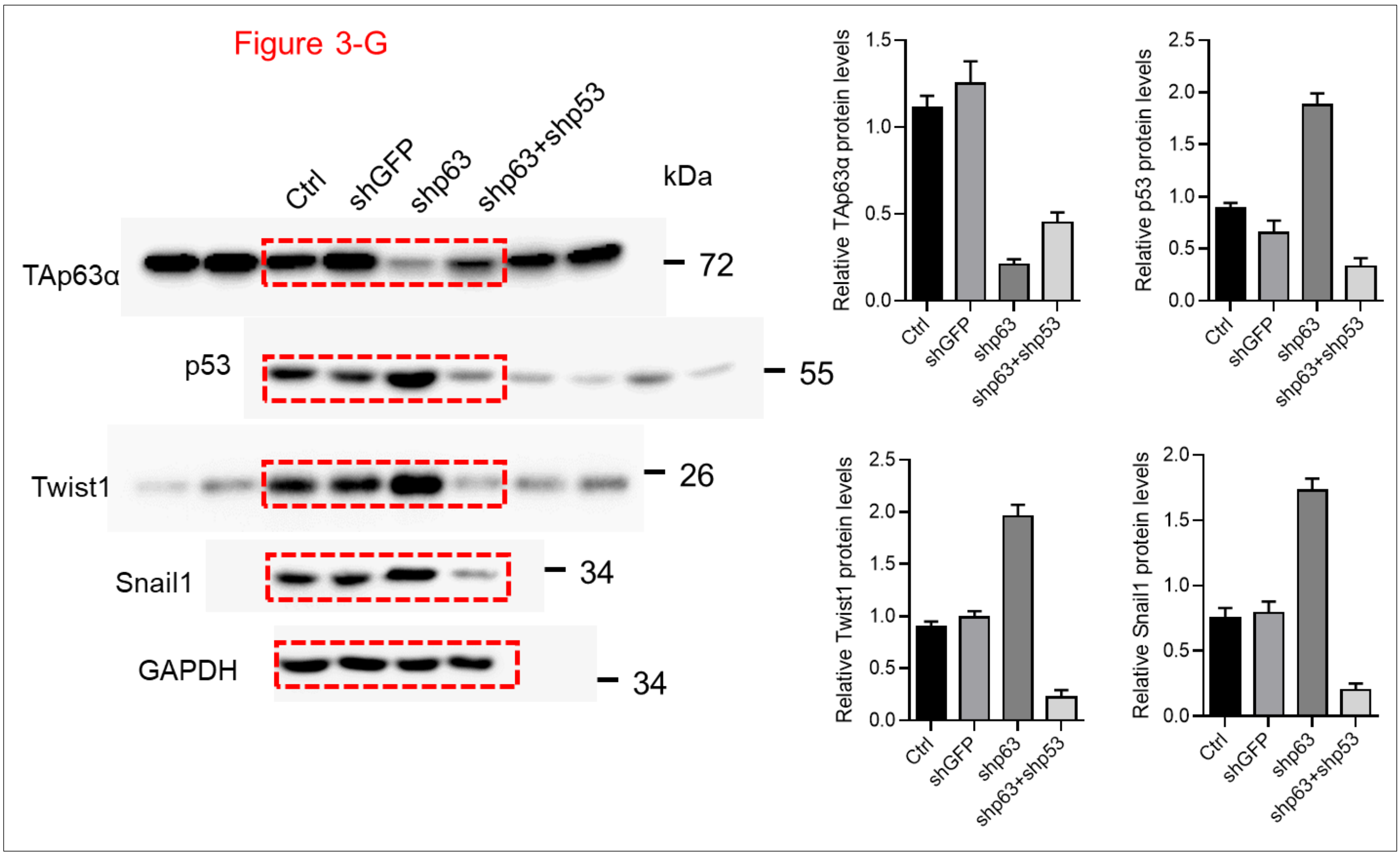


**Figure S14.** knockdown of TAp63α-induced upregulation of Snail1 and twist1 protein expression were totally reversed by simultaneous knockdown of p53-R248W.

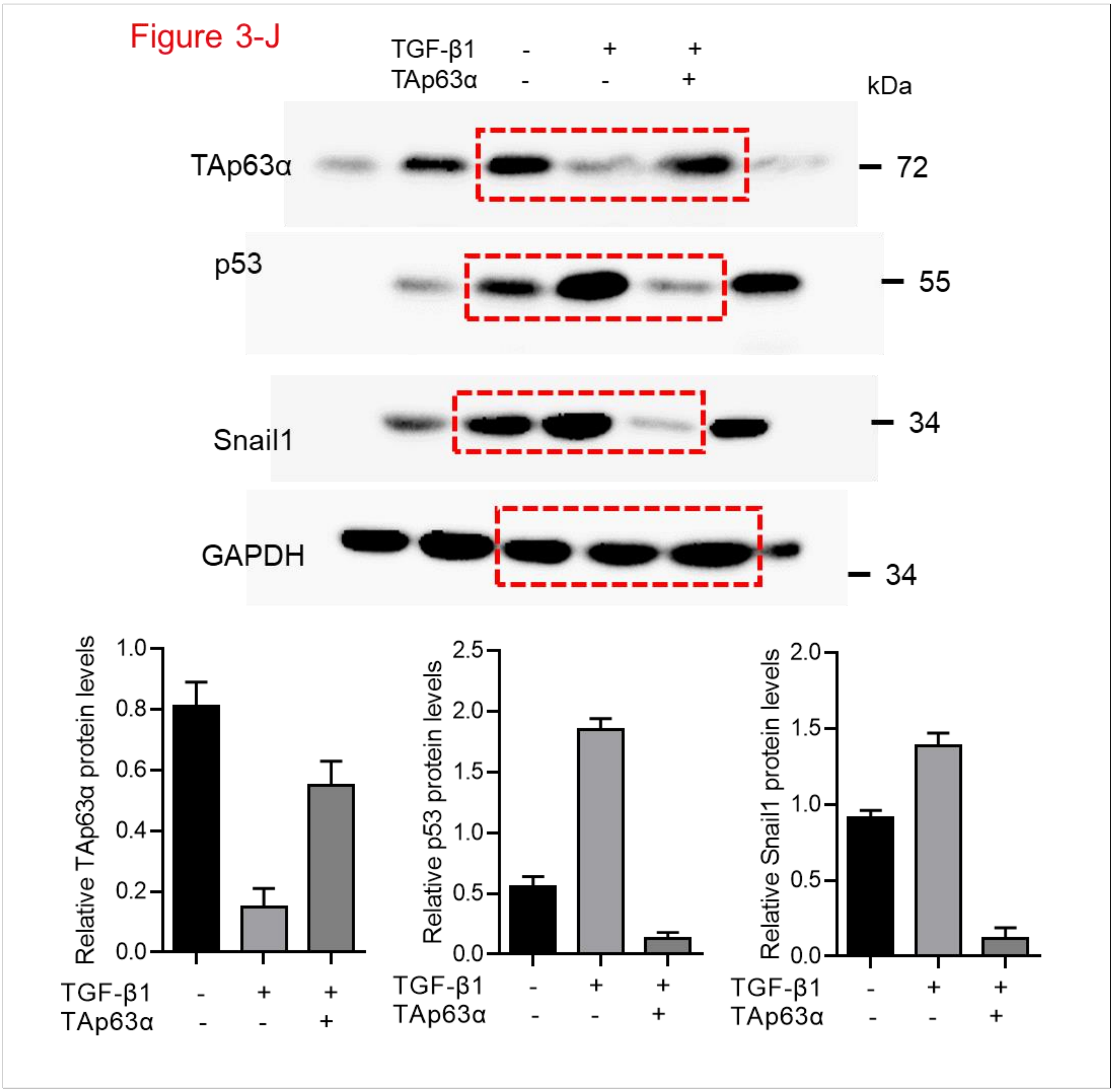


**Figure S15.** Ectopic expression of TAp63α can totally inhibit TGF-β1-induced upregulation of p53-R248W and Snail1 protein expression.

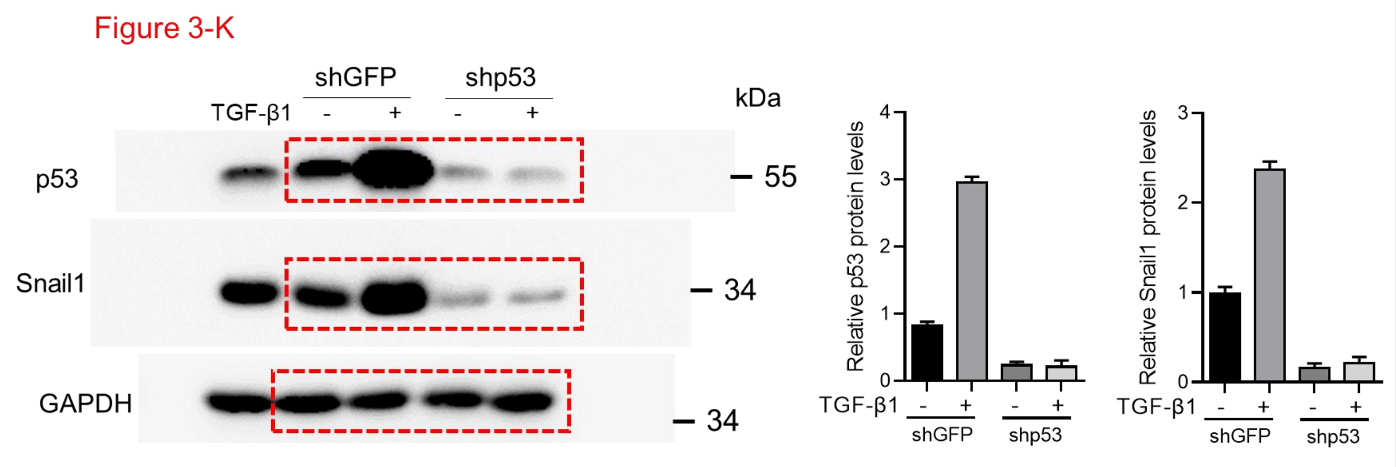


**Figure S16.** TGF-β1-induced snail1 protein expression can be completely suppressed by silencing of p53-R248W expression.
